# Effects of Kiwifruit Peel Extract and Its Antioxidant Potential on the Quality Characteristics of Beef Sausage

**DOI:** 10.3390/antiox11081441

**Published:** 2022-07-25

**Authors:** Evans Frimpong Boateng, Ziyi Yang, Wangang Zhang

**Affiliations:** Key Laboratory of Meat Processing and Quality Control, Ministry of Education China, Jiangsu Collaborative Innovation Center of Meat Production and Processing, Quality and Safety Control, College of Food Science and Technology, Nanjing Agricultural University, Nanjing 210095, China; nanafrimpong94@yahoo.com (E.F.B.); sdfxyzy1998@163.com (Z.Y.)

**Keywords:** kiwifruit peel, agro-waste valorization, beef sausages, antioxidant

## Abstract

In the wake of arresting consumers’ health concerns associated with synthetic antioxidants used in meat products, kiwifruit peel by-product was explored as a natural antioxidant source in the current study. A lyophilized kiwifruit peel extract (KPE) at various concentrations of KPE1 (1.5%), KPE2 (3%), and KPE3 (4.5%) was incorporated into formulated beef sausages to compare the physicochemical, sensory quality, and antioxidant efficacy to the treatments of control (CT 0% KPE) and butylated hydroxytoluene (BHT 0.01%) during 12 d of refrigerated (4 ± 1 °C) storage. The KPE inclusion levels induced significantly higher yellowness (b*) values than CT and BHT, whereas no negative influence of KPE was revealed for lightness (L*) and redness (a*). The pH values of the KPE treatments were reduced, and cooking yield increased significantly (*p* < 0.05), in line with the increasing amount of KPE percentages (1.5%, 3%, and 4.5%) compared to CT and BHT samples. E-nose results showed an enhancement in aroma in KPE treatments, compared to BHT and CT, during the storage period. KPE3 treatment showed a constant lesser value in 2-Thiobarbituric acid reactive substances (TBARS) as storage days increased, compared to the CT and BHT samples. Overall, the KPE is effective for antioxidative capacity, and has the potential to be used as a natural antioxidant in beef sausage.

## 1. Introduction

Meat possesses a high nutritive value in human diet due to the high proportion of protein, caloric fat, vitamins, and microelements needed for the proper function of human metabolic processes [1]. Processed meat commodities, such as sausages, are popular in the world due to their nutritional values and convenience [2]. However, the oxidation of lipid and protein caused by a continuous free-radical chain reaction, cell disruption, oxygen exposure, ultraviolet light, and pro-oxidant eminent vulnerability of meat products, including beef sausages, has presented challenges [3,4,5].

Synthetic antioxidant products have the potential carcinogenicity and toxic effects to pose health risks to consumers [6]. Thus, antioxidant compounds or mixtures from natural sources that extend food products’ shelf life are in high demand [7]. Exploitation of natural antioxidants remains a challenge in the meat industry, for “healthy” meat product development, to avert the health risk concerns associated with synthetic preservative agents [7]. Kiwifruit peel is a by-product secured from processing kiwifruit into slices and nectars, yielding approximately 10–16% of the whole-fruit mass, depending on the peeling method [8]. Indeed, kiwifruits contain many phytonutrients, and several works have reported the presence of polyphenols [9], vitamin C and E [10], flavonoids [11], and other bioactive compounds, thereby confirming their antioxidative properties. Some scientific literature has documented the application of kiwifruit on beef bulgogi textural quality [12], and the effect of proteolytic enzymes purified from kiwifruit on the tenderness of various beef parts [13,14], and food protein digestion [15]. However, research on kiwifruit (edible part) utilization in the meat industry is scarce, particularly regarding kiwifruit by-products (peels, pulp, and seeds). Hence, the high bioactive ingredients and easy accessibility of kiwifruit and its derivatives encouraged the valorization of kiwifruit peels in the current study. Therefore, the present study was aimed at investigating the antioxidant capacity of utilizing lyophilized underutilized KPE, and its effects on beef sausage quality, in comparison with synthetic antioxidant BHT, during refrigerated storage.

## 2. Materials and Methods

### 2.1. Materials

The entire study was conducted in the pilot plant and laboratory of the National Center of Meat Quality and Safety Control, Nanjing Agricultural University (Nanjing, China). Boneless round beef of 24 h postmortem cut and associated fat (subcutaneous fat and inter-muscular fat) were purchased from a supermarket (Suguo Supermarket, Xiamanfang, Nanjing, China) and transported to the laboratory via cold chain. Seasonings in Table 1 used in the beef sausage formulations were supplied by the pilot plant. BHT was acquired from Henan Wanbang Chemical Technology Co., Ltd. (Zhengzhou, China). Chemicals and reagents including 2-thiobarbituric acid (TBA) and trichloroacetic acid (TCA) used in the study were purchased from Sinopharm Chemical Reagent Co., Ltd. (Beijing, China), and all other chemicals employed were of analytical grade.

### 2.2. Production of Lyophilized Kiwifruit Peel Extract

Physiologically matured kiwifruits (*Actinidia deliciosa* cv. Hayward) were sourced from an orchard in Zhouzhi county (Xi’an, China) located at 108°3′50′′ east longitude and 34°17′2′′ north latitude. The fruits were selected, washed, and cleaned with distilled water to eliminate any inorganic material. Peels were separated manually from the edible portions and lyophilized (Christ Lyo Chamber Guard 121550 PMMA, Beijing BMH Instrument Co., Ltd.) at −80 °C for 72 h. The lyophilized peels were manually shredded and ground to a powder by an electrical stainless-steel high-speed multifunction grinder (950 W, Huangdai Instrument Co., Ltd., Shanghai, China). The obtained powder was sieved using a stainless-steel standard inspection sieve with a nominal mesh aperture (0.3 mm) hand sieve shaker (Nanjing Xioncheng Screen Factory, Nanjing, China) to obtain a fine powder. The kiwifruit peel extract was transferred into a plastic bag, sealed, and kept at 4 °C until further use.

### 2.3. Beef Sausage Preparation

The whole sausage products for the study were prepared based on the formulation in Table 1. Five different treatments of sausages, each containing the primary ingredient, were produced with varying percentages of KPE. The control samples (CT) contained 0% KPE, the BHT samples contained 0.01% BHT—based on the permissible range for food additives utilization in China in conformity with national food safety standards [16], KPE1 contained 1.5% KPE, KPE2 contained 3% KPE, and KPE3 contained 4.5% KPE. Firstly, the visible connective tissues were manually trimmed off the meat. Following this, a kitchen blender (JZR-G608, Zhongshan Jinzheng Electrical Appliance Co., Ltd., Zhongshan, China) was used to mince the beef along with associated fat for 3 min. Afterward, water was added and blended for 1 min. Half of the ingredients were added and minced for 2 min. Next, the second half of the ingredients was introduced to obtain a homogenous sausage emulsion after mincing for 2 min. Subsequently, the sausage batter was divided into five groups. The different percentages of BHT and KPE were incorporated, and mixed for 2 min to obtain a homogenate emulsion of beef sausage.

A manual sausage-stuffing machine (Sausage maker Enema machine, Zhongshan, China) was used to manually stuff the sausage batter into a 25 mm diameter synthetic collagen casing (Baan Thai Food Company, Shanghai, China), which was then hand tied to obtain 5 cm-long links, approximately. The beef sausages were subjected to a smoking chamber (T1900 EL 619, FEESMANN, GmbH and Co. KG, 71364 Winnenden, Germany) and smoked at 80 ± 1 °C for 1 h. After smoking, samples were allowed to reach an ambient temperature. Next, after the cooling process, beef sausages were tightly covered by polyethylene film (Surong Plastic Products Co., Ltd., Suzhou, China) and shelved at refrigerated storage (4 ± 1 °C), ahead of later analysis. The experiment was done in three batches on separate days, and each batch was considered as a replicate. Each treatment was randomly selected for analysis at 0, 3, 6, 9, and 12 d.

### 2.4. Color Measurement

A portable colorimeter (CR-400, Konica Minolta, Inc., Tokyo, Japan) with illuminant (D 65), viewing area (0°), and viewing area diameter (0.13 mm) was employed to attain the color of beef sausages after 0, 3, 6, 9, and 12 d of refrigerated storage (4 ± 1 °C). Before measurement, the device was calibrated with a white tile (mod CR-A43) as the standard. Product samples were allowed to bloom color at ambient temperature after being removed from the refrigerated condition. Randomly, eighteen measurements of three replicates from each treatment’s exterior and internal locations were analyzed, and mean value was determined by measuring lightness (L*), redness (a*), and yellowness (b*).

### 2.5. pH Measurement

A digital pH meter (Thermo-Scientific Trion Series, Milan, Italy) adjusted with (pH 4, 7, and 10) buffer solution was used to determine the pH value of beef sausages after 0, 3, 6, 9, and 12 d of refrigerated preservation (4 ± 1 °C).

### 2.6. Cooking Yield Determination

The cooking yield determined in this study adopted the method of Gao et al. [17]. The formula below was employed in computing the correspondence of cooked mass to raw weight values and expounded in percentage:

$$\%\;\mathrm{Cooking}\;\mathrm{yield}=\frac{\mathrm{Cooked}\;\mathrm{weight}}{\mathrm{Raw}\;\mathrm{weight}}\times 100$$

### 2.7. Lipid oxidation Determination

The TBARS were evaluated with slight moderation upon the protocol of Zhang et al. [18]. TBARS values were expressed as mg malondialdehyde (MDA)/kg of meat sample. The samples were evaluated in triplicates.

### 2.8. Electronic Nose

The technique outlined by Zhang et al. [19] was adopted, with slight modification to execute the e-nose analysis of the experimental beef sausages stored at 12 d.

### 2.9. Statistical Analysis

The study considered the completely randomized design (CRD). Three different batches of sausages were prepared at three different days, and each batch was considered as the experimental replication. Five treatment groups (CT, BHT, KPE1, KPE2, and KPE3) of each batch were prepared. The TBtools software (Toolbox for Biologist, v.0.6673; CJ-Chen, South China Agricultural University, Guangzhou, China) was used to draw the merged heatmap of e-nose sensors, and the OriginLab program (Version 12.5; OriginLab Corporation, Northampton, MA, USA) was utilized to perform all analyses. A one-way analysis of variance (ANOVA) and Tukey’s tests were applied in mean values of multiple comparisons among the five treatments, to evaluate significant differences.

## 3. Results and Discussion

### 3.1. Color

Color is regarded as a criterion for meat and meat product purchase by meat consumers. The effects of the various level of KPE, BHT, and CT on exterior and internal color are shown in Table 2. The thermal processing of products in the smoke chamber revealed a significant interaction which altered the surface color of products L*, a*, and b* significantly, compared to internal color. In addition, the surface a* reduced value during storage demonstrated that products a* were storage-duration dependent. The results of the color measurement showed a significant difference (*p* < 0.05) in L*, a*, and b* of samples across the 12 d of storage. The L* values in KPE1- and KPE2-treated samples were reduced significantly throughout storage compared to CT and BHT. The lower values observed were percentage dependent (1.5%, 3%, and 4.5%) for the KPE in the beef sausage products. Lightness in food products (meat) is influenced by many determinants, including the concentration and the pigment type in plant extract, the chemical type of the pigment, and the meat composition [20]. Similarly, a* values were less in the KPE treatments compared to CT and BHT. The lesser changes in the product’s surface L*, compared to internal, is linked to dominance in brightness reflection intensity from the product’s inner part, and the product’s external opacity, caused by the thermal process. Comparatively, fluctuating values were recorded in KP1 and KPE3 treatments on d 6 and 12, which may be subjected to oxidation effect. Hence, the findings of a decrease in a* can be ascribed to the existence of non-meat ingredients in the KPE, and the polyphenolic compounds present in the KPE treatments. In addition, the L* and a* values were not significantly different (*p* > 0.05) in CT treatments, across storage duration. Therefore, the color alterations of BHT and KPE treatments may be related to catalyzed iron atoms during KPE pigment oxidation or denaturation of myoglobin (Mb) molecules by cooking temperature [21]. A significant increase (*p* < 0.05) in b* value was revealed in KPE samples compared to CT and BHT surface and inner color due to the inclusion of KPE concentration in the beef sausages. Owing to the diverse score recorded in all samples, desirable L*, a*, and b* surface and inner color factors indicated the stability of the samples’ color traits, regardless of the slight discoloration in redness in the KPE treatments. Hence, the color variation was attributed to the KPE concentration incorporated in the beef sausage.

### 3.2. pH Evaluation

The pH value of KPE treatments was significantly lower (*p* < 0.05) compared to CT and BHT samples during storage, as shown in Table 3. On d 6, the KPE samples showed a significant pH decline compared to the control. The high content of protein, fat, and water in meat products makes the meat medium favorable for microbial growth and spoilage speed [22]. Therefore, the lower pH of meat products generates an acidic medium, leading to lower water-holding capacity (WHC) with increased cooking and drip losses [23]. The pH and ionic strength of the meat system can greatly influence the WHC through protein and water interaction [24]. The pH results are in line with similar significantly reduced pH results of meat products treated with moringa leaf powder during the storage period [25], flaxseed, tomato powder [26], and destoned olive cake powder [27]. Hence, the low pH after KPE treatments implies that KPE may enhance the pH stability of beef sausages.

### 3.3. Cooking Yield

In general, the results revealed a significant difference (*p* < 0.05) in cooking yield in KPE compared to CT and BHT samples during storage (Table 4). Lesser values were recorded on the initial day, but a higher decline in values was realized after d 3 in CT and BHT samples. In contrast, all the KPE-treated samples had consistently high cooking yield values proportional to the increased KPE percentage in beef sausages. On the other hand, the cooking yield values comparatively indicated no significant difference (*p* < 0.05) in KPE treatments until d 6. This observation was due to the dissipation of moisture in CT and BHT samples during cooking (thermal process). This may be due to the ability of KPE to interact in the sausages’ matrix to hold inner water and bind the protein gel matrix, resulting in protein denaturation inhibition [28]. In addition, Gao et al. [2] reported that moisture evaporation and fat-melting during the thermal process yield cooking losses. This finding corroborates with [29] and confirms the binding ability of plant extract, which increased the stability characteristics in meat products treated with fruit by-products after cooking.

### 3.4. TBARS

**Figure 1:**
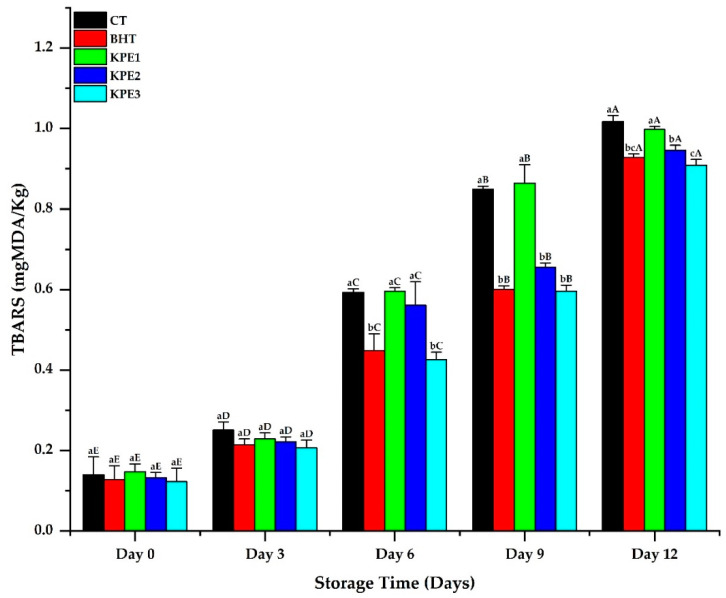
TBARS results of beef sausages during 12 d of refrigerated storage. Values are mean ± SE (n = 3). ^a–c^ Means values are significantly different (*p* < 0.05) across the treatments, while ^A–E^ means values are significantly different (*p* < 0.05) across the storage duration. CT: (0% KPE), BHT: (0.01%), KPE1: (1.5%), KPE2: (3%), and KPE3: (4.5%) stored at 4 ± 1 °C for 12 d.

According to Zhang et al. [19] and Li and Liu [30], lipid oxidation contributes to meat products’ deterioration in quality, due to compounds produced after free radicals’ chain reactions. Figure 1 shows the production of TBARS in stored beef sausages. Significant changes were observed in all the treatments, predominantly on d 6 of storage, as d 0 and 3 values revealed slight variations. This slight stability evidenced the deficient level of secondary oxidative products in the early days of storage. Instead, a significant change (*p* < 0.05) was observed in CT, BHT, and KPE treatments on d 9 and 12 of storage, leading to a steady rise in the treatments’ TBARS values. This signifies hydro-peroxide mechanisms, which result in higher TBARS formation [31]. However, the TBARS values of the KPE samples were lower compared to the CT sample, relative to the increasing storage days. The higher TBARS values of the CT samples evidence a higher level of lipid oxidation, which can significantly cause deterioration in the beef sausage’s quality. Lipid content and composition of fatty acid connect to oxidative stability directly, and in that process, a high lipid and polyunsaturated fatty acids (PUFA)/saturated fatty acid (SFA) ratio increases meat lipid oxidation [32]. Hence, the result of higher oxidation products revealed in the CT samples is in agreement with the positive correlation between fat content and lipid oxidation [33]. In addition, the oxidation inhibition of KPE treatments may be attributed to flavonoids and phenolic content, which scavenge the oxidation process of free radicals that initiate auto-oxidation by contributing a hydrogen atom from a phenolic hydroxyl group (-OH) [34].

In contrast, the TBARS values of BHT treatments were slightly lower than KPE1 and KPE2 treatments. This may have resulted from a considerable ineffectiveness of the low level of the KPE in the beef sausage. According to Kalogianni et al. [35], diverse antioxidants exhibited varying thresholds based on pro-oxidant and meat matrix. In addition, Khan et al. [16] reported that natural antioxidants exhibited different potency in meat products stored under various storage conditions. However, TBARS values of KPE3 treatment showed a lesser value with increasing storage days, when compared to the BHT samples. This reveals that the inhibitory effect of KPE against lipid peroxidation was dose-dependent, with 4.5% KPE demonstrating the least TBARS value.

### 3.5. Electronic Nose

**Figure 2:**
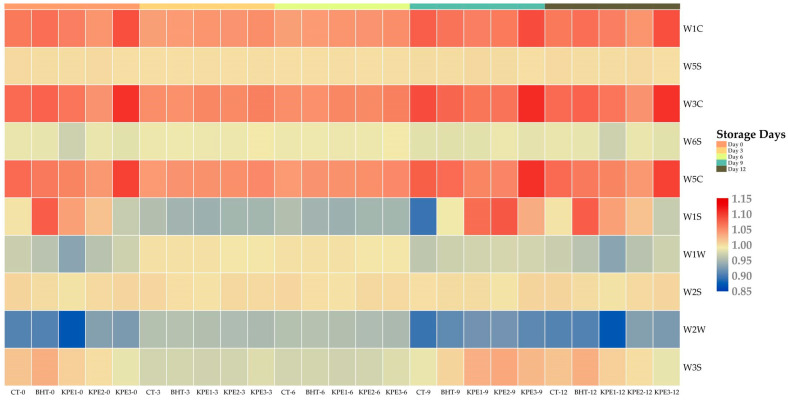
Heatmap of E-nose sensor responses in beef sausages during 12 d of refrigerated storage. CT: (0% KPE), BHT: (0.01%), KPE1: (1.5%), KPE2: (3%), and KPE3: (4.5%) stored at 4 ± 1 °C for 12 d.

Figure 2 presents e-nose results of beef sausages, and Table 5 indicates the chemical sensor (10) array response. A significant difference (*p* < 0.05) was noticed based on the increased e-nose values with storage days. On d 0, a statistical difference (*p* < 0.05) was seen in CT samples compared to BHT and KPE treatments. The response tendency of CT samples exhibited a fluctuating sensor sensitivity value in (W1S) and (W3S) sensitivity to methane and aliphatic, respectively. However, the WIS response from the e-nose of the CT sample was inconsistent on d 0 and d 12 on the heatmap, which may be associated with oxidizing gas emission suppression, since volatile organic compounds (VOCs) contain both unstable reducing and oxidizing agents. In addition, different responses in volatile substance sensors showed no significant differences (*p* > 0.05) in BHT and KPE treatment at d 3 and d 6 of storage. All treatments responded less to sensitivity towards (W2W) aromatic sulfur compounds at d 3 and d 6 of storage, with d 0, 9, and 12 indicating slight variation in sensors’ sensitivities. Water-soluble compositions and lipids are the two substantive precursors of meat flavor. Therefore, Milliard reaction between amid amino and carbonyl compounds, reducing sugar, and lipid breakdown products during the heat process and pathways interactions yield aroma volatility [36]. Hence, this may be a possible factor in the (W2W) result. Further, no sensitivity detection of (W1S) methane in the environment with a broad range was present in CT, BHT, and KPE samples from d 0 to d 6, until d 9. Yet, W1S was slightly higher in BHT samples, but less in KPE treatments, comparatively. This corresponds to the degree of KPE percentage in the beef sausage products. The BHT and KPE treatments affected (W1C) sensitivity to aromatic compounds, (W5S) sensitivity to nitrogen oxides, (W3C) sensitivity to ammonia and aromatic compounds, (W6S) sensitivity to hydrogen, (W5C) sensitivity to alkenes and aromatic compounds, (W1W) sensitivity to sulfur compounds, and (W2S) sensitivity to alcohols and a broad range of incompletely aromatic compounds, demonstrated by the heatmap color dynamics. In contrast, a fluctuating sensitivity of CT samples to W3S was detected. Lipid oxidation produces several hundred VOCs, including aldehydes, alcohol, aliphatic hydrocarbons, ketones, esters, and carboxylic acids [37]. Therefore, Maillard reaction and the oxidation of unsaturated fatty acid generate volatile compound in meat, thereby contributing to meat flavor and odor development [38]. The mechanism of KPE impacted the sensory trait of beef sausages due to changes revealed in the aroma profile. The result reveals that e-nose was dependent on W1C, W5S, W5C, W3C, W1W, and W2S sensors, indicating the presence of several VOCs, higher in KPE compared to CT and BHT samples. Finally, samples’ sensor responses exhibited a varying and stable VOCs intensity in storage days at different response patterns. However, a positive relation of e-nose pathway in KPE treatments exhibited a high trend for a positive role in aroma characteristics, compared to CT and BHT samples.

## 4. Conclusions

Findings from this present study revealed that KPE did not negatively influence the L* and a* color values, yet the b* values were higher compared to CT and BHT, due to KPE pigment oxidation. In addition, KPE lowered pH values, and the cooking yield results were improved for the KPE treatments compared to CT and BHT treatments, due to KPE’s ability to interact by holding inner water and binding with the sausage protein gel matrix. KPE retarded lipid oxidation of beef sausages due to phenolic compound in KPE. The KPE treatments contributed to a higher aroma profile from e-nose results than CT and BHT treatments, which had a high VOC presence in KPE. Conclusively, KPE addition may improve the quality characteristics of beef sausages. However, further study of the antimicrobial effect of KPE on beef sausage safety is recommended.

## Figures and Tables

**Table 1:** Beef sausages formulation.

Ingredients	Sample Groups Amount (%)				
	CT	BHT	KPE1	KPE2	KPE3
Meat	77.83	77.82	76.33	74.83	73.33
Added fat	7.00	7.00	7.00	7.00	7.00
Mixed spices	1.25	1.25	1.25	1.25	1.25
Garlic powder	0.80	0.80	0.80	0.80	0.80
Onion powder	1.20	1.20	1.20	1.20	1.20
NaNO_2/_NaNO_3_	0.12	0.12	0.12	0.12	0.12
Red pepper powder	0.50	0.50	0.50	0.50	0.50
Black pepper powder	0.50	0.50	0.50	0.50	0.50
NaCl	1.25	1.25	1.25	1.25	1.25
Water	9.55	9.55	9.55	9.55	9.55
BHT	-	0.01	-	-	-
KPE	-	-	1.50	3.00	4.50
**Total**	**100**	**100**	**100**	**100**	**100**

KPE: lyophilized kiwifruit peel extract, BHT: butylated hydroxytoluene, CT: Control (0% KPE), BHT (0.01% BHT), KPE1 (1.5% KPE), KPE2 (3% KPE), KPE3 (4.5% KPE), NaCl: Sodium chloride, NaNO_2_: Sodium nitrite and NaNO_3_: Sodium nitrate.

**Table 2:** Color of beef sausages during 12 d of refrigerated storage.

Storage Time (Days)	Sample Groups External Color				
	CT	BHT	KPE1	KPE2	KPE3
	L*				
Day 0	53.99 ± 3.40 ^aA^	53.47 ± 2.03 ^aA^	52.52 ± 3.27 ^aA^	48.99 ± 3.31 ^bA^	47.69 ± 3.03 ^bA^
Day 3	53.48 ± 2.38 ^aA^	53.38 ± 1.35 ^aA^	52.46 ± 3.43 ^aA^	48.51 ± 3.89 ^bA^	47.44 ± 3.59 ^bA^
Day 6	53.53 ± 1.34 ^aA^	53.22 ± 3.20 ^aA^	50.05 ± 2.74 ^bAB^	47.61 ± 4.32 ^cA^	46.57 ± 3.83 ^dA^
Day 9	52.97 ± 2.01 ^aA^	52.96 ± 2.47 ^aA^	48.93 ± 4.20 ^bB^	46.27 ± 3.89 ^cAB^	44.95 ± 2.12 ^dA^
Day 12	48.87 ± 1.22 ^aB^	47.39 ± 1.35 ^bB^	44.59 ± 2.50 ^cC^	43.94 ± 2.88 ^dB^	39.89 ± 2.09 ^eB^
	a*				
Day 0	9.68 ± 1.70 ^aA^	8.87 ± 2.51 ^bA^	7.95 ± 2.27 ^cA^	5.83 ± 1.60 ^dA^	5.32 ± 1.74 ^dA^
Day 3	9.36 ± 2.40 ^aAB^	8.64 ± 2.15 ^bA^	7.62 ± 2.19 ^cA^	5.64 ± 1.06 ^dAB^	5.27 ± 1.00 ^dA^
Day 6	9.24 ± 1.85 ^aB^	7.96 ± 1.85 ^bB^	7.23 ± 2.05 ^cAB^	5.40 ± 1.54 ^dB^	5.07 ± 1.10 ^eB^
Day 9	8.98 ± 1.76 ^aC^	7.81 ± 1.51 ^bB^	6.82 ± 1.85 ^cB^	5.23 ± 1.40 ^dB^	4.83 ± 1.22 ^eB^
Day 12	8.47 ± 2.35 ^aD^	7.46 ± 2.29 ^abB^	6.79 ± 2.23 ^bAB^	5.11 ± 1.18 ^cB^	4.62 ± 1.28 ^dB^
	b*				
Day 0	11.82 ± 2.08 ^abA^	11.40 ± 1.31 ^bA^	12.04 ± 1.61 ^abA^	12.26 ± 1.47 ^aA^	12.35 ± 1.42 ^aA^
Day 3	11.29 ± 1.18 ^bAB^	11.34 ± 1.58 ^bA^	11.44 ± 1.75 ^bAB^	12.32 ± 1.54 ^aA^	12.29 ± 1.99 ^aA^
Day 6	11.25 ± 1.49 ^cAB^	11.19 ± 1.44 ^dAB^	11.39 ± 2.03 ^bB^	11.97 ± 1.90 ^aA^	12.10 ± 1.88 ^aA^
Day 9	10.95 ± 1.42 ^cB^	10.65 ± 1.02 ^dB^	11.34 ± 1.14 ^bB^	11.84 ± 1.67 ^aA^	11.90 ± 1.59 ^aA^
Day 12	9.98 ± 1.48 ^dC^	9.93 ± 2.11 ^eC^	10.30 ± 1.63 ^cC^	10.66 ± 1.97 ^bB^	10.80 ± 1.62 ^aB^
	**Sample Groups Internal Color**				
	L*				
Day 0	55.73 ± 2.00 ^aA^	55.14 ± 3.80 ^aA^	54.23 ± 4.35 ^aA^	53.27 ± 3.56 ^bA^	52.39 ± 3.50 ^cA^
Day 3	55.01 ± 1.79 ^aA^	54.73 ± 3.17 ^aA^	54.68 ± 3.33 ^aA^	53.15 ± 3.26 ^bA^	52.51 ± 3.93 ^cA^
Day 6	55.76 ± 2.90 ^aA^	53.87 ± 1.53 ^bB^	53.12 ± 3.07 ^bB^	52.95 ± 2.71 ^cA^	51.94 ± 4.48 ^dB^
Day 9	55.64 ± 2.39 ^aA^	53.17 ± 2.28 ^bB^	52.10 ± 4.48 ^cB^	49.03 ± 4.78 ^bB^	48.34 ± 4.88 ^bC^
Day 12	50.79 ± 4.47 ^aB^	47.44 ± 2.63 ^bC^	46.44 ± 4.98 ^cC^	45.63 ± 4.13 ^dC^	42.91 ± 4.45 ^eD^
	a*				
Day 0	10.54 ± 2.01 ^aA^	9.54 ± 1.73 ^bA^	8.53 ± 1.57 ^cA^	6.15 ± 1.54 ^dA^	5.86 ± 1.29 ^dA^
Day 3	10.29 ± 1.39 ^aAB^	9.39 ± 2.17 ^bA^	8.46 ± 1.67 ^cA^	5.82 ± 1.09 ^dAB^	5.81 ± 1.04 ^dA^
Day 6	10.19 ± 2.78 ^aB^	8.63 ± 1.95 ^bB^	7.45 ± 1.50 ^cAB^	5.56 ± 1.92 ^dB^	5.52 ± 1.10 ^dB^
Day 9	9.68 ± 1.25 ^aC^	8.52 ± 1.60 ^bB^	7.05 ± 1.51 ^cB^	5.61 ± 1.48 ^dB^	5.28 ± 1.22 ^eB^
Day 12	9.28 ± 2.50 ^aD^	8.28 ± 2.00 ^abB^	7.31 ± 1.02 ^bAB^	5.47 ± 1.27 ^cB^	5.47 ± 1.04 ^cB^
	b*				
Day 0	9.74 ± 1.88 ^abA^	9.53 ± 2.03 ^bA^	11.39 ± 1.94 ^abA^	11.67 ± 1.70 ^aA^	11.84 ± 1.93 ^aA^
Day 3	9.59 ± 1.70 ^bAB^	9.47 ± 1.35 ^bA^	11.17 ± 1.50 ^abAB^	11.77 ± 1.90 ^aA^	11.79 ± 1.80 ^aA^
Day 6	9.48 ± 2.34 ^cAB^	9.18 ± 1.84 ^dAB^	10.68 ± 1.96 ^bB^	11.37 ± 2.03 ^aA^	11.63 ± 2.21 ^aA^
Day 9	9.05 ± 1.54 ^cB^	9.03 ± 1.86 ^dB^	10.63 ± 2.39 ^bB^	11.26 ± 1.78 ^aA^	11.57 ± 2.36 ^aA^
Day 12	8.95 ± 1.83 ^dC^	8.85 ± 1.54 ^eC^	10.04 ± 2.18 ^cC^	10.53 ± 2.66 ^abB^	11.02 ± 2.57 ^aB^

Values are mean ± SD (n = 18). ^a–e^ Means values are significantly different (*p* < 0.05) across the treatments, while ^A–D^ means values are significantly different (*p* < 0.05) across the storage duration. CT: (0% KPE), BHT: (0.01%), KPE1: (1.5%), KPE2: (3%), and KPE3: (4.5%) stored at 4 ± 1 °C for 12 d.

**Table 3:** pH values of beef sausages during 12 d of refrigerated storage.

Storage Time (Days)	pH				
	CT	BHT	KPE1	KPE2	KPE3
Day 0	6.58 ± 0.05 ^aA^	6.50 ± 0.01 ^bA^	6.18 ± 0.01 ^cA^	6.14 ± 0.03 ^dA^	5.88 ± 0.01 ^eA^
Day 3	6.43 ± 0.01 ^aC^	6.42 ± 0.04 ^aB^	6.15 ± 0.02 ^bB^	6.10 ± 0.02 ^cB^	5.81 ± 0.06 ^dAB^
Day 6	6.48 ± 0.01 ^aB^	6.30 ± 0.01 ^bC^	5.96 ± 0.01 ^cC^	5.88 ± 0.04 ^dC^	5.63 ± 0.02 ^eB^
Day 9	6.20 ± 0.02 ^aD^	6.17 ± 0.06 ^bD^	5.78 ± 0.31 ^cD^	5.63 ± 0.29 ^dD^	5.59 ± 0.07 ^eBC^
Day 12	5.85 ± 0.06 ^aE^	5.76 ± 0.30 ^bE^	5.45 ± 0.33 ^dE^	5.51 ± 0.27 ^cE^	5.35 ± 0.08 ^eC^

Values are mean ± SE (n = 3). ^a–e^ Means values are significantly different (*p* < 0.05) across the treatments, while ^A–E^ means values are significantly different (*p* < 0.05) across the storage duration. CT: (0% KPE), BHT: (0.01%), KPE1: (1.5%), KPE2: (3%), and KPE3: (4.5%) stored at 4 ± 1 °C for 12 d.

**Table 4:** Cooking yield of beef sausages during 12 d of refrigerated storage.

Storage Time (Days)	Cooking Yield (%)				
	CT	BHT	KPE1	KPE2	KPE3
Day 0	95.06 ± 1.68 ^bA^	95.01 ± 0.25 ^bA^	97.74 ± 0.38 ^abA^	98.75 ± 0.45 ^abA^	98.95 ± 0.05 ^aA^
Day 3	93.74 ± 0.20 ^cA^	93.02 ± 0.40 ^cAB^	97.51 ± 0.22 ^bA^	98.65 ± 0.01 ^aA^	98.67 ± 0.14 ^aA^
Day 6	89.18 ± 0.06 ^bB^	88.91 ± 2.26 ^bB^	93.15 ± 0.01 ^abB^	96.18 ± 0.01 ^aB^	96.83 ± 1.31 ^aB^
Day 9	88.57 ± 0.70 ^bB^	88.29 ± 0.97 ^bB^	92.71 ± 0.21 ^abC^	93.19 ± 0.34 ^abC^	94.13 ± 2.48 ^aC^
Day 12	87.80 ± 4.57 ^cB^	87.48 ± 0.67 ^cC^	90.71 ± 4.12 ^bD^	93.55 ± 3.65 ^aC^	93.60 ± 0.17 ^aC^

Values are mean ± SE (n = 3). ^a–c^ Means values are significantly different (*p* < 0.05) across the treatments, while ^A–D^ means values are significantly different (*p* < 0.05) across the storage duration. CT: (0% KPE), BHT: (0.01%), KPE1: (1.5%), KPE2: (3%), and KPE3: (4.5%) stored at 4 ± 1 °C for 12 d.

**Table 5:** Information on 10 chemical sensor array responses.

Chemical Sensor	Chemical Sensor Class	Descriptions
W1S	Broad methane	Sensitive to methane
W5S	Broad range	Sensitive to nitrogen oxides
W1W	Sulfur organics	Sensitive to organic sulfides
W6S	Hydrogen	Sensitive to hydrogen compounds
W3S	Methane aliphatic	Sensitive to methane and aliphatic
W3C	Aromatic	Sensitive to ammonia, aromatic molecules
W2S	Broad alcohols	Sensitive to alcohols, ketones, and aldehydes
W1C	Aromatic	Sensitive to aromatic and benzene compounds
W2W	Sulfur chloride	Sensitive to organic-sulfides and organic-chloride
W5C	Aromatic aliphatic	Sensitive to methane, propane, and aliphatic non-polar molecules

## Data Availability

Not applicable.
